# Comparative Efficacy of Endoscopic Versus Open Surgical Techniques in the Management of Gastric Outlet Obstruction: A Systematic Review

**DOI:** 10.7759/cureus.73690

**Published:** 2024-11-14

**Authors:** Shenouda Abdallah, Muath M Dabas, Rami K Morcos, Abdur Rehman, Abdullah Shehryar, Asif Orakzai, Manukrishna Sivadasan, Talha A Zia, Nabila N Anika, Nada B Abushalha, Syed Jameel

**Affiliations:** 1 Surgery, Jaber Al-Ahmad Hospital, Kuwait, KWT; 2 Surgery, The University of Jordan, Amman, JOR; 3 General Surgery, Ain Shams University Specialized Hospital, Cairo, EGY; 4 General Surgery, Ministry of Health Holdings, Dammam, SAU; 5 Surgery, Mayo Hospital, Lahore, PAK; 6 Internal Medicine, Allama Iqbal Medical College, Lahore, PAK; 7 Ophthalmology, Rehman Medical Institute, Peshawar, PAK; 8 Surgery, Jawaharlal Institute of Postgraduate Medical Education and Research, Puducherry, IND; 9 Internal Medicine, King Edward Medical University, Lahore, PAK; 10 Surgery, Baylor College of Medicine, Houston, USA; 11 Medicine and Surgery, Holy Family Red Crescent Medical College Hospital, Dhaka, BGD; 12 Gynecology, The University of Jordan, Amman, JOR

**Keywords:** comparative efficacy, endoscopic ultrasound-guided gastroenterostomy, gastric outlet obstruction, gastrojejunostomy, systematic review

## Abstract

Gastric outlet obstruction (GOO) is a clinical condition that can arise from both benign and malignant causes, requiring effective management strategies to ensure optimal patient outcomes. Traditionally, open surgical techniques like gastrojejunostomy (GJ) have been the standard treatment, but recent advances in minimally invasive procedures, such as endoscopic ultrasound-guided gastroenterostomy (EUS-GE), offer alternative approaches with potentially reduced morbidity. This systematic review compared the efficacy, safety, and clinical outcomes of endoscopic versus open surgical techniques in managing GOO. A comprehensive search of major electronic databases, including PubMed, MEDLINE, Embase, the Cochrane Library, and Scopus, identified relevant studies published from January 2014 to September 2024. The analysis included randomized controlled trials, clinical trials, and meta-analyses involving a total of 8,540 patients. Results indicated that EUS-GE showed high technical and clinical success rates (91-94% and 88-89.9%, respectively) and lower complication rates (6.8-13.1%) compared to open surgical approaches, which were associated with higher perioperative risks but demonstrated better long-term outcomes in specific scenarios, such as malignant GOO. The findings suggest that while endoscopic techniques are preferable for patients with high surgical risk, open surgery may still be necessary in complex cases. Further research, including randomized controlled trials and long-term studies, is recommended to refine these strategies and improve clinical decision-making. This review underscores the importance of tailored treatment approaches in optimizing the management of GOO, balancing efficacy, safety, and patient-centered outcomes.

## Introduction and background

Gastric outlet obstruction (GOO) is a clinical syndrome characterized by the impaired passage of gastric contents into the proximal small intestine, which can arise from a variety of benign and malignant conditions [1]. Traditionally, the management of GOO has relied on open surgical techniques, which, while effective, are associated with significant morbidity and mortality rates, particularly in patients with malignant obstructions or in those with poor surgical candidacy due to comorbid conditions [2]. In recent years, the advent of endoscopic techniques, particularly endoscopic ultrasound-guided gastroenterostomy (EUS-GE), has offered a less invasive alternative, promising reduced recovery times and complications [3,4]. However, the comparative efficacy and safety of these endoscopic techniques against traditional open surgical methods, such as gastrojejunostomy (GJ), remain subjects of ongoing clinical research and debate [5]. This systematic review aims to synthesize current evidence on the outcomes of these two divergent approaches, providing a comprehensive overview of their respective efficacies in the management of GOO.

The primary objective of this systematic review is to compare the efficacy and safety profiles of endoscopic techniques, notably EUS-GE, against open surgical techniques for the management of GOO. By evaluating a range of outcomes, including technical success rates, clinical efficacy, adverse events, and long-term patient survival, this review seeks to delineate the contexts in which each approach may be most beneficial. The review will also consider patient-centered outcomes such as recovery times, quality of life, and the need for subsequent interventions, which are crucial for optimizing treatment strategies in diverse patient populations. Through rigorous analysis of contemporary data, this systematic review intends to guide clinical decision-making and inform future therapeutic directions in the treatment of GOO.

## Review

Materials and methods

Search Strategy

Our search strategy was carefully designed following the Preferred Reporting Items for Systematic Reviews and Meta-Analyses (PRISMA) guidelines [6] to identify comparative studies on endoscopic and open surgical techniques for the management of GOO. Comprehensive searches were conducted in key electronic databases including PubMed, MEDLINE, Embase, the Cochrane Library, and Scopus, with the timeframe restricted to the last decade, from January 2014 to September 2024. This period was chosen to focus on the most recent advancements and outcomes related to these surgical interventions.

Keywords and Medical Subject Headings (MeSH) were selected to specifically address the core aspects of our research question. The terms included "gastric outlet obstruction", "endoscopic ultrasound-guided gastroenterostomy", "gastrojejunostomy", "surgical outcomes", "clinical efficacy", and "minimally invasive surgical techniques". Boolean operators ('AND', 'OR') were employed to structure and optimize the search. Example search strings were as follows: "gastric outlet obstruction AND endoscopic techniques", "gastrojejunostomy OR surgical outcomes AND comparative efficacy", and "minimally invasive surgical techniques AND GOO management". To ensure comprehensive coverage and capture any potentially relevant unpublished or ongoing studies, we also explored reference lists of selected articles, relevant conference proceedings, and clinical trial registries. This methodical approach aims to encompass a broad spectrum of literature, providing a robust foundation for evaluating the comparative efficacy of these surgical techniques in treating GOO.

Eligibility Criteria

The eligibility criteria for this systematic review are rigorously defined to ensure the inclusion of studies that are both relevant and of high methodological quality. Our review focuses on peer-reviewed research articles, including clinical trials, randomized controlled trials, and meta-analyses, that explore the outcomes of endoscopic versus open surgical techniques in the management of GOO. To ensure a comprehensive analysis, we include studies that provide data on clinical outcomes, technical success rates, postoperative complications, and long-term patient survival associated with each surgical approach.

Inclusion criteria are specifically tailored to select studies that offer a direct comparison between endoscopic and open surgical interventions for GOO. We consider studies published within the last 10 years to reflect recent advancements and practices in medical treatment. Only articles published in English and in peer-reviewed journals are included to ensure the quality and accessibility of the data. We specifically look for studies that provide clear descriptions of patient demographics, intervention details, and standardized outcome measures. Exclusion criteria include studies focusing on non-comparative, single-technique assessments, non-human research, narrative reviews, and case reports. Additionally, studies that do not provide sufficient detail on the methodology or outcome measures, as well as those reporting on non-malignant causes of GOO without differentiation, are also excluded to maintain the focus and quality of the review. This strategic selection is designed to ensure that the conclusions drawn from the systematic review are based on robust and high-quality evidence directly applicable to clinical decision-making in the treatment of GOO.

Data Extraction

Our data extraction process was carefully crafted to ensure precision and thoroughness in gathering data for our systematic review on the comparative efficacy of endoscopic versus open surgical techniques in managing GOO. Initially, each article identified through our search strategy was assessed by two independent reviewers based on titles and abstracts to determine its relevance to our research focus. This initial filtration was critical to pinpointing articles that warranted more detailed examination.

Following this preliminary selection, articles classified as potentially relevant underwent full-text review. During this phase, data extraction was performed using a customized data extraction template in Microsoft Excel (Microsoft Corporation, Redmond, Washington, United States) to standardize the process and minimize errors. Each reviewer independently recorded essential details from each study, including the publication year, study design, sample size, primary outcomes, key findings, and any noted methodological limitations. Discrepancies between reviewers were resolved through consultation with a third, senior reviewer to ensure consistency and objectivity in our data compilation. This methodical approach facilitated a meticulous aggregation of data, crucial for the subsequent synthesis and analysis phases of our review, aiming to provide clear insights into the optimal surgical interventions for GOO.

Data Analysis and Synthesis

Our data analysis and synthesis adopted a qualitative approach due to the heterogeneity in study designs, interventions, and outcomes across the selected studies. This method allowed us to systematically assess the comparative efficacy of endoscopic and open surgical techniques for managing GOO. We categorized the findings from each study to identify patterns and discrepancies in key outcomes, such as technical success rates, complication rates, recovery times, and long-term survival. Through narrative synthesis, we integrated these findings to provide a comprehensive understanding of the advantages and limitations of each surgical approach, discussed their implications for clinical practice, and highlighted gaps in the current literature that warrant further investigation.

Results

Study Selection Process

**Figure 1:**
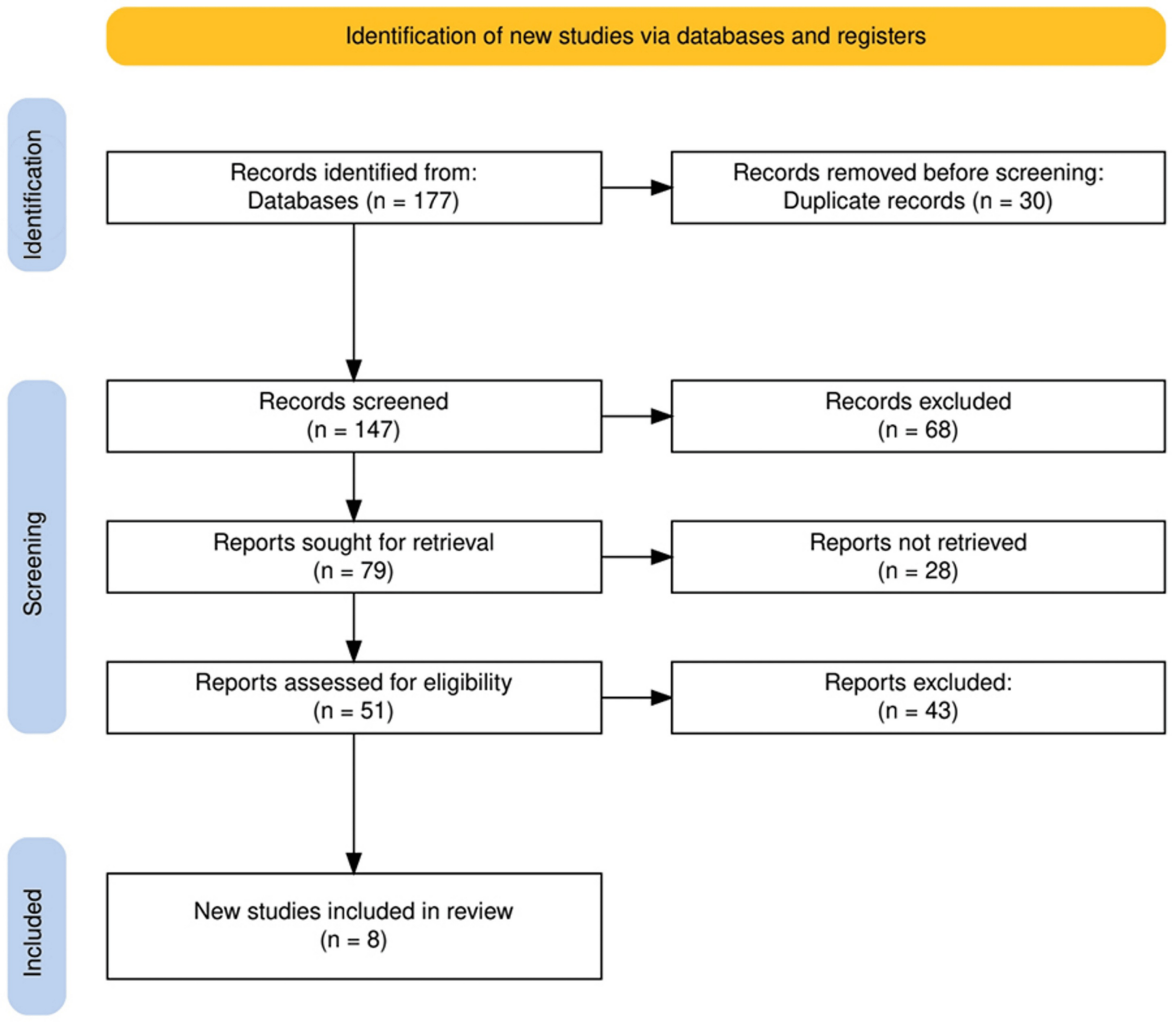
The PRISMA flowchart represents the study selection process. PRISMA: Preferred Reporting Items for Systematic Reviews and Meta-Analyses

The study selection process for this systematic review followed a structured approach to ensure the inclusion of relevant and high-quality studies. Initially, a total of 177 records were identified through comprehensive searches of various electronic databases. After removing 30 duplicate records, 147 records remained for screening. These records were then screened based on their titles and abstracts, resulting in the exclusion of 68 records that did not meet the inclusion criteria. For the remaining 79 records, full-text retrieval was attempted, but 28 reports could not be retrieved. Of the 51 reports assessed for eligibility, 43 were excluded based on predefined exclusion criteria, such as not directly comparing endoscopic and open surgical techniques for GOO. Ultimately, eight new studies were included in the review, providing a robust foundation for a comprehensive analysis of the comparative efficacy and safety of the different management approaches for GOO. A summary of the whole study selection process is given in Figure 1.

Characteristics of Selected Studies

The selected studies for this systematic review include a mix of meta-analyses, systematic reviews, and clinical trials, all focused on evaluating different surgical and endoscopic techniques for managing GOO. These studies provide a comprehensive overview of the efficacy, safety, and clinical outcomes associated with various approaches, such as EUS-GE, GJ, and the use of covered versus uncovered self-expandable metal stents (C-SEMSs vs. U-SEMSs). Collectively, the studies encompass diverse patient populations and clinical scenarios, offering valuable insights into the strengths and limitations of each technique and helping guide clinical decision-making for optimal patient management (Table 1).

**Table 1:** Summary of the key studies discussed in the article. EUS-GE: endoscopic ultrasound-guided gastroenterostomy; GOO: gastric outlet obstruction; AEs: adverse events; EUS-GE vs. SGE: endoscopic ultrasound-guided gastroenterostomy versus surgical gastroenterostomy; EUS-GE vs. ES: endoscopic ultrasound-guided gastroenterostomy versus endoscopic stenting; C-SEMSs: covered self-expanding metal stents; U-SEMSs: uncovered self-expanding metal stents; mGOO: malignant gastric outlet obstruction; ORs: odds ratios; SGJ: surgical gastrojejunostomy; RRs: risk ratios; ES: endoscopic stenting; GJ: gastrojejunostomy; MGOO: malignant gastric outlet obstruction; OS: overall survival; JT/GT: jejunal tube/gastrostomy tube; EUS-GEA: endoscopic ultrasound-guided gastrojejunostomy with LAMS; LAMS: lumen-apposing metal stents; PRISMA: Preferred Reporting Items for Systematic Reviews and Meta-Analyses; PCSEMS: partially covered self-expanding metal stents; LAG: laparoscopic anastomosis; GOOSS: Gastric Outlet Obstruction Scoring System; CENTRAL: Cochrane Central Register of Controlled Trials

Authors	Study type	Objective	Materials and methods	Results	Conclusion
Li et al., 2023 [7]	Comprehensive meta-analysis	Evaluate the efficacy and safety of EUS-GE for GOO	Review of PubMed, Embase, Web of Science, and Cochrane Library to identify relevant studies	26 studies included, with 1493 patients. High technical (94%) and clinical success rates (89.9%). AEs: 13.1%. Subgroup comparisons show EUS-GE vs. SGE and EUS-GE vs. ES with various ORs for technical and clinical success and AEs	EUS-GE is effective and minimally invasive for GOO
Tringali et al., 2020 [8]	Systematic review and meta-analysis	Compare the efficacy of C-SEMSs vs. U-SEMSs in mGOO	Review of MEDLINE, Embase, and Cochrane Library from 2000 to 2019 to identify studies comparing C-SEMSs versus U-SEMSs	16 studies included, with 1741 patients. Higher stent survival with C-SEMSs. No significant difference in patient survival. Various ORs for clinical and technical success, AEs, stent occlusion, and migration	Further studies on C-SEMSs with antimigration systems are recommended
Fan et al., 2022 [9]	Systematic review and meta-analysis	Determine the clinical outcomes of EUS-GE for GOO	Keyword search "EUS-guided gastroenterostomy" in PubMed, Web of Science, and Cochrane databases	10 studies, 297 patients managed with EUS-GE. High technical (91%) and clinical success rates (88%). Complications rate: 6.8%. Comparisons with SGJ showed various RRs for success and complications	EUS-GE has a lower complication rate and is a safe, effective, and minimally invasive choice for managing GOO
Khamar et al., 2023 [10]	Systematic review and meta-analysis	Comprehensively evaluate ES vs. GJ for the palliation of malignant GOO	Review of MEDLINE, Embase, and CENTRAL databases for comparative studies of adult patients undergoing ES or GJ. Primary and secondary outcomes measured	39 full-text articles, 3128 ES patients, and 2116 GJ patients included. Shorter survival in ES, fewer complications, quicker recovery, but higher reintervention risk	Both ES and GJ are appropriate in select clinical scenarios
Wang et al., 2023 [11]	Systematic review and Bayesian network meta-analysis	Compare the OS and treatment outcomes of GJ in MGOO	Search of PubMed, Embase, Web of Science, and CENTRAL up to 2022. Bayesian network meta-analysis performed	24 retrospective studies, 2473 patients. GJ demonstrated the most effective treatment in terms of OS. Improved subsequent anticancer treatment, ranking second only to JT/GT	GJ improves OS and follow-up treatments in MGOO, supporting its selection as an appropriate therapy
Antonelli et al., 2020 [12]	Systematic review and meta-analysis	Provide a pooled estimate of technical and clinical outcomes for EUS-GEA using LAMS	Search conducted in PubMed, Embase, Scopus, and Web of Science databases until February 2019. PRISMA methodology applied	12 studies, 290 patients included. High technical (93.5%) and clinical success rates (90.1%). Pooled total adverse events rate was 11.7%	EUS-GEA is a safe, effective, minimally invasive alternative to surgery
Yamao et al., 2021 [13]	Randomized controlled trial	Compare the efficacy of CSEMS vs. UCSEMS in mGOO	Multicenter randomized prospective study. Main outcomes were stent dysfunction and patient survival, with subgroup analyses	No significant difference in OS. Higher dysfunction rate in UCSEMS, especially for extrinsic tumors. Lower tumor ingrowth in CSEMS for intrinsic tumors and higher migration rates for extrinsic tumors	Subgroup analysis suggests covered stents for intrinsic and uncovered for extrinsic malignancies
Chung et al., 2016 [14]	Clinical trial	Evaluate PCSEMS for managing benign anastomotic strictures post-LAG	Retrospective analysis of PCSEMS placement for benign obstruction after LAG between May 2007 and June 2012	11 patients included. Successful stent placement and improved GOOSS scores. No major complications. Stent dysfunction occurred in four patients	PCSEMS is a feasible and effective option to avoid secondary surgery post-LAG

Discussion

The systematic review reveals distinct differences in efficacy, technical success rates, complication rates, and patient outcomes between endoscopic and open surgical techniques for managing GOO. EUS-GE, as examined in multiple studies, demonstrated high technical success rates ranging from 91% to 94% and clinical success rates between 88% and 89.9%, with a relatively low overall complication rate of 6.8-13.1%. In comparison, open surgical techniques such as GJ showed variable outcomes; while GJ improved overall survival (OS) and supported subsequent anticancer treatments in malignant GOO cases, it was associated with a longer recovery period and a higher risk of surgical complications. Studies comparing C-SEMSs vs. U-SEMSs also highlighted notable differences: C-SEMSs provided better stent survival but at the cost of higher migration rates, whereas U-SEMSs had a higher risk of stent occlusion but lower migration rates.

This review suggests that while endoscopic techniques like EUS-GE offer a less invasive option with fewer complications and faster recovery times, open surgical approaches such as GJ may be more effective in certain clinical scenarios, particularly when longer-term outcomes or complex malignant cases are considered. These findings underscore the importance of individualized treatment planning, considering patient-specific factors such as the nature of the obstruction, comorbid conditions, and overall surgical risk, to optimize clinical outcomes in managing GOO.

The findings of our systematic review largely align with the existing literature that underscores the advantages of minimally invasive techniques, such as EUS-GE, in managing GOO [15]. Prior studies have consistently reported high technical and clinical success rates for EUS-GE, with lower complication rates compared to open surgical approaches like GJ [16]. Our review corroborates these findings, highlighting the effectiveness and safety of EUS-GE, particularly in reducing postoperative recovery time and minimizing adverse events. This is in agreement with studies, which also reported favorable outcomes for EUS-GE in patients with both benign and malignant GOO [17]. However, our review provides a more comprehensive comparison by incorporating a broader range of studies and patient populations, including the elderly and those with significant comorbidities, thereby offering a more nuanced understanding of when EUS-GE might be preferred over traditional surgical options [18].

In contrast, the role of open surgical techniques like GJ remains more debated in the current literature. While some studies have reported GJ as superior in terms of long-term survival, especially in patients with malignant GOO [19], our findings suggest that this advantage may come at the cost of higher postoperative morbidity and longer recovery periods. For example, a study by Khashab et al. [16] found that while GJ provided durable relief from obstruction, it was associated with higher complication rates compared to endoscopic methods. Our review extends these observations by demonstrating that the choice between endoscopic and open surgical techniques should be tailored to individual patient profiles, considering factors such as tumor type and location and patient overall health status. These nuanced insights contribute significantly to the ongoing discourse by highlighting the need for a more personalized approach to managing GOO, balancing the benefits of minimally invasive techniques with the potential long-term advantages of traditional surgery in specific clinical contexts [20].

The findings of this systematic review have several practical implications for clinical practice in managing GOO. Endoscopic techniques, particularly EUS-GE, may be preferable in patients with significant comorbidities or those deemed high-risk for open surgery due to its minimally invasive nature, high technical and clinical success rates, and lower complication rates [21]. This approach can lead to faster recovery times and reduced hospital stays, making it an ideal choice for elderly patients or those with poor overall health. Conversely, open surgical techniques such as GJ might be more suitable in cases of severe or recurrent malignant obstruction where longer-term patency and outcomes are prioritized, despite the higher immediate postoperative risks [22]. The evidence suggests that clinicians should consider individual patient characteristics, such as the severity of obstruction, presence of comorbid conditions, and overall functional status, when choosing the most appropriate intervention. By aligning the choice of technique with specific clinical scenarios, healthcare providers can optimize treatment outcomes, minimize complications, and enhance the quality of life for patients with GOO.

This systematic review's strengths lie in its comprehensive search strategy and rigorous inclusion criteria, which ensured the inclusion of a broad range of high-quality studies on endoscopic and open surgical techniques for managing GOO. By employing a systematic approach in line with the PRISMA guidelines, we minimized selection bias and ensured a robust analysis of both recent and seminal studies across multiple databases. However, several limitations must be acknowledged. The heterogeneity of the included studies, in terms of study design, patient populations, and outcome measures, posed challenges in directly comparing findings across studies. Additionally, the reliance on published literature may introduce publication bias, as studies with negative or inconclusive results are less likely to be published. Furthermore, some studies lacked detailed reporting on patient subgroups and specific surgical techniques, which could affect the generalizability of our findings to all clinical settings. These limitations highlight the need for a cautious interpretation of the results and suggest areas for future research to address these gaps and refine clinical guidelines for GOO management.

The differences observed between endoscopic and open surgical techniques in managing GOO can be attributed to their distinct mechanistic approaches and procedural invasiveness [23]. Endoscopic techniques, such as EUS-GE, tend to result in fewer complications due to their minimally invasive nature, which reduces trauma to surrounding tissues and lowers the risk of infection, bleeding, and postoperative pain [24]. This minimally invasive approach also allows for a quicker recovery and shorter hospital stay, particularly beneficial for patients with comorbidities or those at higher surgical risk. In contrast, open surgical techniques like GJ often demonstrate higher technical success rates, particularly in complex cases involving severe obstructions or malignancies, due to the surgeon's direct visualization and ability to manage intricate anatomical challenges. However, the increased invasiveness of open surgery inherently carries a higher risk of perioperative complications, including infections and longer recovery times. Understanding these mechanistic differences is crucial in guiding clinicians to select the most appropriate technique based on patient-specific factors, such as the severity of obstruction, underlying pathology, and overall health status [25].

Based on the findings of this systematic review and the gaps identified in the existing literature, several recommendations for future research are warranted. There is a clear need for more robust, randomized controlled trials comparing endoscopic techniques such as EUS-GE with traditional open surgical methods like GJ in diverse patient populations, particularly focusing on long-term outcomes and quality of life [26]. Further comparative studies should also investigate the efficacy and safety of C-SEMSs vs. U-SEMSs in various clinical contexts to better understand their role in managing malignant GOO. Additionally, long-term follow-up studies are essential to evaluate the durability of different interventions and their impact on survival, recurrence rates, and the need for reinterventions [27]. Research focusing on patient-centered outcomes, such as functional status, recovery times, and overall patient satisfaction, would also be valuable in guiding clinical decision-making. Addressing these areas will not only fill current knowledge gaps but also refine treatment strategies and improve outcomes for patients with GOO.

## Conclusions

This systematic review highlights the nuanced differences in the efficacy, safety, and clinical outcomes of endoscopic versus open surgical techniques for managing GOO. Endoscopic approaches, particularly EUS-GE, demonstrate a favorable profile with high technical and clinical success rates and lower complication rates, making them a viable option for patients at higher surgical risk or with significant comorbidities. Conversely, open surgical techniques like GJ offer potential advantages in specific scenarios, particularly for malignant obstructions, but are associated with higher perioperative morbidity and longer recovery times. These findings underscore the importance of personalized treatment planning in clinical practice, considering patient-specific factors and the nature of the obstruction. However, there remains a need for further high-quality, randomized controlled trials and long-term follow-up studies to better delineate the optimal use of these techniques across different patient populations. By advancing our understanding of these surgical options, future research can refine treatment strategies and ultimately improve patient outcomes in the management of GOO.

## References

[1] Kumar A, Annamaraju P (2024). Gastric outlet obstruction. StatPearls [Internet].

[2] Potz BA, Miner TJ (2016). Surgical palliation of gastric outlet obstruction in advanced malignancy. World J Gastrointest Surg.

[3] Wang G, Liu X, Wang S, Ge N, Guo J, Sun S (2019). Endoscopic ultrasound-guided gastroenterostomy: a promising alternative to surgery. J Transl Int Med.

[4] Kastelijn JB, Moons LM, Garcia-Alonso FJ (2020). Patency of endoscopic ultrasound-guided gastroenterostomy in the treatment of malignant gastric outlet obstruction. Endosc Int Open.

[5] Tyberg A, Perez-Miranda M, Sanchez-Ocaña R (2016). Endoscopic ultrasound-guided gastrojejunostomy with a lumen-apposing metal stent: a multicenter, international experience. Endosc Int Open.

[6] Page MJ, McKenzie JE, Bossuyt PM (2021). The PRISMA 2020 statement: an updated guideline for reporting systematic reviews. BMJ.

[7] Li JS, Lin K, Tang J, Liu F, Fang J (2023). EUS-guided gastroenterostomy for gastric outlet obstruction: a comprehensive meta-analysis. Minim Invasive Ther Allied Technol.

[8] Tringali A, Costa D, Anderloni A, Carrara S, Repici A, Adler DG (2020). Covered versus uncovered metal stents for malignant gastric outlet obstruction: a systematic review and meta-analysis. Gastrointest Endosc.

[9] Fan W, Tan S, Wang J (2022). Clinical outcomes of endoscopic ultrasound-guided gastroenterostomy for gastric outlet obstruction: a systematic review and meta-analysis. Minim Invasive Ther Allied Technol.

[10] Khamar J, Lee Y, Sachdeva A (2023). Gastrojejunostomy versus endoscopic stenting for the palliation of malignant gastric outlet obstruction: a systematic review and meta-analysis. Surg Endosc.

[11] Wang C, Zhang X, Liu Y, Lin S, Yang C, Chen B, Li W (2023). Efficacy and long-term prognosis of gastrojejunostomy for malignant gastric outlet obstruction: a systematic review and Bayesian network meta-analysis. Eur J Surg Oncol.

[12] Antonelli G, Kovacevic B, Karstensen JG, Kalaitzakis E, Vanella G, Hassan C, Vilmann P (2020). Endoscopic ultrasound-guided gastro-enteric anastomosis: a systematic review and meta-analysis. Dig Liver Dis.

[13] Yamao K, Kitano M, Chiba Y (2021). Endoscopic placement of covered versus uncovered self-expandable metal stents for palliation of malignant gastric outlet obstruction. Gut.

[14] Chung KH, Lee SH, Park JM (2016). Partially covered self-expandable metallic stent for postoperative benign strictures associated with laparoscopy-assisted gastrectomy. Gastric Cancer.

[15] Asghar M, Forcione D, Puli SR (2024). Endoscopic ultrasound-guided gastroenterostomy versus enteral stenting for gastric outlet obstruction: a systematic review and meta-analysis. Therap Adv Gastroenterol.

[16] Khashab MA, Bukhari M, Baron TH (2017). International multicenter comparative trial of endoscopic ultrasonography-guided gastroenterostomy versus surgical gastrojejunostomy for the treatment of malignant gastric outlet obstruction. Endosc Int Open.

[17] McCarty TR, Garg R, Thompson CC, Rustagi T (2019). Efficacy and safety of EUS-guided gastroenterostomy for benign and malignant gastric outlet obstruction: a systematic review and meta-analysis. Endosc Int Open.

[18] Carbajo AY, Kahaleh M, Tyberg A (2020). Clinical review of EUS-guided gastroenterostomy (EUS-GE). J Clin Gastroenterol.

[19] Cheung SL, Teoh AY (2022). Optimal management of gastric outlet obstruction in unresectable malignancies. Gut Liver.

[20] Sibio S, La Rovere F, Di Carlo S (2022). Benefits of minimally invasive surgery in the treatment of gastric cancer. World J Gastroenterol.

[21] Conti Bellocchi MC, Gasparini E, Stigliano S (2024). Endoscopic ultrasound-guided gastroenterostomy versus enteral stenting for malignant gastric outlet obstruction: a retrospective propensity score-matched study. Cancers (Basel).

[22] Nagaraja V, Eslick GD, Cox MR (2014). Endoscopic stenting versus operative gastrojejunostomy for malignant gastric outlet obstruction-a systematic review and meta-analysis of randomized and non-randomized trials. J Gastrointest Oncol.

[23] Jeong SJ, Lee J (2020). Management of gastric outlet obstruction: focusing on endoscopic approach. World J Gastrointest Pharmacol Ther.

[24] Bomman S, Ghafoor A, Sanders DJ, Jayaraj M, Chandra S, Krishnamoorthi R (2022). Endoscopic ultrasound-guided gastroenterostomy versus surgical gastrojejunostomy in treatment of malignant gastric outlet obstruction: systematic review and meta-analysis. Endosc Int Open.

[25] Singh SS, Shinde RK (2023). Minimally invasive gastrointestinal surgery: a review. Cureus.

[26] Ribas PH, De Moura DT, Proença IM (2022). Endoscopic ultrasound-guided gastroenterostomy for the palliation of gastric outlet obstruction (GOO): a systematic review and meta-analysis of the different techniques. Cureus.

[27] Papanikolaou IS, Siersema PD (2022). Gastric outlet obstruction: current status and future directions. Gut Liver.

